# Roles of parental stress and children’s emotional skills on behavioral responses: evidence from NSCH parent reports

**DOI:** 10.3389/fpsyg.2025.1525077

**Published:** 2025-07-22

**Authors:** Fa Zhang, Chin-Chih Chen, Yuyan Xia, Yaoying Xu, Jamie Cage

**Affiliations:** ^1^School of Education, Open University of China, Beijing, China; ^2^School of Education, Virginia Commonwealth University, Richmond, VA, United States; ^3^Department of Physical Therapy, University of Kentucky, Lexington, KY, United States; ^4^School of Social Work, Virginia Commonwealth University, Richmond, VA, United States

**Keywords:** self-regulation, parental stress, emotional skills, behavioral responsiveness, preschool, parent reports

## Abstract

**Introduction:**

Early self-regulation is a crucial predictor of how well preschoolers respond to challenging, frustrating, and distracting situations. Examining the mediating role of contextual and individual factors provides insight into how external influences and personal characteristics shape children’s ability to navigate these challenges. This study aims to investigate whether specific contextual (e.g., parental stress) and individual (e.g., emotional skills) factors mediate the relationship between preschoolers’ self-regulation and their behavioral responses to transitions, frustration, and distraction.

**Methods:**

We utilized data from the 2021 National Survey of Children’s Health, which includes a nationally representative sample of children aged 3–5 in the U.S. (*N* = 11,554). Structural equation modeling was employed to examine the mediating effects of parental stress (contextual factor) and emotional skills (individual factor) on the association between preschoolers’ self-regulation and their behavioral responsiveness.

**Results:**

The analysis revealed that higher levels of self-regulation were associated with more positive emotional skills, lower parental stress, and improved behavioral responsiveness in children. Among the mediators, parental stress significantly mediated the relationship between preschoolers’ self-regulation and behavioral responsiveness, while emotional skills did not show a significant mediating effect.

**Discussion:**

These findings suggest that contextual factors, particularly parental stress, play a more substantial mediating role than individual emotional skills in shaping the relationship between self-regulation and behavioral responses in preschoolers. This highlights the importance of addressing parenting-related stress in intervention programs aimed at supporting children’s behavioral development.

## 1 Introduction

Research on children’s behavioral responses to transitions, frustration, and distraction is highly valued in early childhood education due to its significant impact on later cognitive and affective success, as well as learning performance upon entering school (Cole et al., 2011; Kahle et al., 2021). This line of research spans various domains, including anxiety, learning difficulties, distractibility, and aggression (Harrison et al., 2012), collectively defined as a broad spectrum of behaviors exhibited by children during transitions, frustration, and distracting situations (Achenbach et al., 2016; Steenhoff et al., 2021). It has been well documented that self-regulation is one of the most significant factors influencing children’s behavioral responses to unpleasant circumstances (Aksan and Kochanska, 2004; Blair and Diamond, 2008; Hill et al., 2006; Li et al., 2023). Self-regulation, also referred to as self-control or self-management in academic discourse, involves the intentional use of one’s abilities to appropriately respond to environmental demands (Kochanska et al., 2001; Ponitz et al., 2008, 2009).

Although the connection between children’s self-regulation and behavioral development is well established, the pathways through which contextual and individual factors influence this relationship require further examination (McClelland and Cameron, 2012; Montroy et al., 2014, 2016). Externally, factors such as parental involvement (Cucu Ciuhan, 2021; Polat and Bayındır, 2022), parenting practices (Harvey et al., 2011), teaching practices (Plueck et al., 2015; Vo et al., 2012), and broader societal influences (e.g., socioeconomic status and ethnic identity, Blair et al., 2011) contribute to children’s behavioral responses. Internally, influences include children’s physical health and motor development (Mancini et al., 2018), problem-solving skills (Fantuzzo et al., 2004), and emotional skills (Grewal et al., 2006; Housman, 2017). Within the framework of ecological systems theory (Bronfenbrenner, 1979), factors affecting children’s behavior and development extend beyond the individual child. Research also suggests that children’s behaviors are shaped by their closest environments, such as family dynamics (Criss et al., 2002) and parental stress (Crum and Moreland, 2017; Giannotti et al., 2022). Thus, while children’s self-regulation is known to be linked to their behavioral development and responsiveness, the specific mechanisms underlying this relationship require further exploration, particularly to understand the concurrent influence of family-related (contextual) and child-related (individual) affective mediating factors.

Grounded in developmental frameworks such as Denham’s model of emotional competence and Trentacosta and Fine’s mediation model of social–emotional development, emotional skills are conceptualized as an affective bridge between self-regulation and behavior. These frameworks support the hypothesis that emotional competence may mediate the effect of self-regulatory capacities on behavioral responsiveness, particularly in social or emotionally charged contexts (Denham et al., 2003; Trentacosta and Fine, 2010).

Recent studies on children’s self-regulation have developed models examining the factors that influence children’s behavioral responses and the mechanisms underlying these processes, often using national datasets (Claussen et al., 2021; Ghandour et al., 2019; Kriegel et al., 2021). National surveys provide critical insights into children’s social, emotional, and behavioral development. Understanding how these systems interact within diverse family contexts is essential for collecting developmentally appropriate, individually relevant, and contextually responsive data to inform effective practices and interventions. Grounded in ecological systems theory (Bronfenbrenner, 1979) and a conceptual framework previously proposed by the authors (Xu et al., 2025), this study utilizes the National Survey of Children’s Health (NSCH), a nationally representative dataset, to examine the contextual and individual factors influencing children’s behavioral responses to transitions, frustration, and distraction. Specifically, this study aims to investigate the role of children’s self-regulation in their behavioral responses to challenging, frustrating, and distracting situations by assessing whether individual emotional skills and/or contextual parental stress mediate this relationship in a representative sample of U. S. children aged 3–5 years.

### 1.1 Self-regulation and children’s behavioral responsiveness

Self-regulation plays a crucial role in shaping how children respond behaviorally to challenging, frustrating, and distracting situations (Doan et al., 2012; Eisenberg et al., 2017; Hill et al., 2006). Children who struggle to control impulsive behaviors in these circumstances often experience both concurrent (Al Otaiba and Fuchs, 2002; Malecki and Elliot, 2002) and subsequent learning difficulties (Masten et al., 2005). Higher-order constructs of self-regulation, such as executive function, attentional control, and effortful control, effectively mitigate disruptive and hyperactive behaviors when children encounter setbacks (Hardaway et al., 2012; Lonigan et al., 2017). While much of the existing research has focused on specific diagnostic groups (Alderson et al., 2007; Willcutt et al., 2005), self-regulation is also associated with variations in children’s behaviors and responses both concurrently (Willoughby et al., 2011) and over time (Hughes and Ensor, 2011; Sulik et al., 2015) among typically developing children.

Higher levels of self-regulation promote children’s psychological well-being and positive emotions, reducing the likelihood of depressive and anxiety disorders as well as somatic complaints (Eisenberg et al., 2010; Watters and Wojciak, 2020). This is particularly important, as children who struggle with transitions in early childhood are at risk for persistent or escalating behavioral difficulties over time (Sterba et al., 2007), which may be linked to long-term mental health challenges (Suominen et al., 2004). These connections have been demonstrated in studies involving both preschool-aged children (Bater and Jordan, 2017; Eisenberg et al., 2017) and older children (Lonigan et al., 2017; Sawyer et al., 2015). Furthermore, early self-regulation skills may have lasting effects on children’s behavioral development, with higher levels of self-regulation at age 4 predicting greater behavioral responsiveness at home and in school throughout primary school (McClelland et al., 2018; Sawyer et al., 2015).

Given the strong evidence linking children’s self-regulation to their behavioral responsiveness, researchers have proposed a more complex mechanism to better understand the affective pathways through which self-regulation and behavioral responsiveness may be associated, particularly by examining potential mediating factors.

*H1*: *Children’s self-regulation is positively correlated with their behavioral responsiveness during transitions, frustration, and distracting situations*.

Affective indicators serve as a key mechanism underlying the relationship between children’s self-regulation and behavioral responsiveness. This study focuses on two aspects of affective indicators: children’s emotional skills and parental stress. As outlined earlier, ecological systems theory suggests that factors influencing children’s behaviors include both individual and contextual elements. This distinction becomes particularly relevant when considering how parental stress (a contextual factor) and children’s emotional skills (an individual factor) interact to shape behavioral development.

### 1.2 A potential individual mediator: emotional skills

Broadly defined in early childhood literature, emotional skills involve the ability to effectively understand, interpret, regulate, and express emotions, as well as respond to the emotions of others (Denham et al., 2012; Shields et al., 2001). As an affective indicator, children’s emotional skills play a crucial role in shaping variations in their behaviors (Castro et al., 2018; Krause et al., 2020; Maguire et al., 2016). The Center on the Social Emotional Foundations for Early Learning defines early emotional skills as a child’s evolving ability, from birth through age five, to establish close and secure relationships with adults and peers, regulate and express emotions in socially and culturally appropriate ways, and explore the environment to acquire knowledge (Yates et al., 2008). Neuroscientific research in child development has identified ages 0–6 as a critical period for the development of emotional skills (Housman et al., 2018). While self-regulation and emotional competence are interrelated, emotional skills represent a distinct socio-emotional domain that influences children’s behavioral responses in interpersonal contexts (Housman, 2017; Korucu et al., 2022; Russell et al., 2016).

Early childhood and the school years mark a crucial period of growth for both behavioral and emotional skills, with a well-documented association between the two. Previous research has shown that a lack of emotional skills in children significantly contributes to behavioral issues, as reduced positive emotions can lead to increased behavioral challenges and poorer responses (Eisenberg et al., 2001; Kim et al., 2007; Silk et al., 2003). Recent findings also indicate that weaker emotional skills are a significant predictor of increased internalizing behaviors in subsequent years (Castro et al., 2018) and that children’s struggles with managing emotions influence their behavioral responsiveness (Crespo et al., 2017).

Several empirical studies have supported emotional skills as mediating variables between self-regulation and children’s behavioral or social outcomes (Eisenberg et al., 2003; Shields et al., 2001; Trentacosta and Izard, 2007). These findings suggest that emotional understanding and regulation may help children apply their self-regulatory capacities in socially appropriate ways, thereby promoting adaptive behavioral responsiveness. Additionally, while cross-sectional (Claussen et al., 2021), longitudinal (Edossa et al., 2018), and intervention studies (Housman et al., 2018) have demonstrated a positive relationship between children’s self-regulation and emotional skills, limited research has focused on the specific pathways linking children’s emotional skills with self-regulation and behavioral responsiveness. In selecting emotional skills as a mediator, we aimed to capture the socio-emotional interpretive processes that may link self-regulation to behavioral outcomes. This addition acknowledges that emotional skills, while situated at the same individual level as self-regulation, offer a distinct pathway by which children make sense of and respond to social experiences. Therefore, the following hypothesis is proposed:

*H2: Children’s emotional skills mediate the relationship between self-regulation and behavioral responsiveness. Specifically, children’s self-regulation is positively correlated with emotional skills (H2a), and children’s emotional skills are positively correlated with behavioral responsiveness (H2b).*

### 1.3 A potential contextual mediator: parental stress

Concerning parental stress, one aspect of the Family Stress Model suggests that exposure to stressful parenting events leads to increased parental stress (Peterson et al., 2010). This psychological distress, in turn, exacerbates children’s behavioral difficulties. Empirical support for this model is found in intervention studies (Crum and Moreland, 2017) and longitudinal research (Osborne and Reed, 2009). Additionally, children’s behavioral responsiveness issues also contribute to heightened parental stress, regardless of whether the children have disabilities (Clauser et al., 2021; Enea and Rusu, 2020) or are typically developing (Baker et al., 2002).

The bidirectional relationship between parental stress and children’s behavioral responsiveness issues has been well-documented (Chiang and Bai, 2024; Krahé et al., 2015), with some studies specifically highlighting the effects of maternal parental stress (Fang et al., 2024; Giannotti et al., 2022). In relation to children’s self-regulation, research findings have been mixed. Some studies report no significant relationship between children’s self-regulation and later parental stress (Planalp et al., 2022), whereas others suggest that stronger self-regulation skills enable children to engage in higher-quality interactions with parents, thereby reducing parental stress and fostering more appropriate behavioral responses (Haslam et al., 2019).

We focused on parenting stress as a contextual mediator based on both empirical evidence and theoretical relevance. Its proximal nature directly affects the parent–child dyad, influencing behavioral regulation, emotional climate, and socialization practices within the family system. This makes it a theoretically compelling mechanism through which broader contextual factors may impact child outcomes. Therefore, drawing on prior literature and ecological systems theory, this study examines how these factors interact through the family mediator, parental stress, and proposes the following hypothesis:

*H3: Parental stress mediates the relationship between children’s self-regulation and behavioral responsiveness. Specifically, children’s self-regulation is inversely correlated with parental stress (H3a), and parental stress is inversely correlated with children’s behavioral responsiveness (H3b).*

### 1.4 NSCH dataset

The National Survey of Children’s Health (NSCH) is the most comprehensive nationwide survey assessing the physical and mental well-being of children aged 0–17 in the U. S. Conducted annually by the U. S. Census Bureau since 2016, the NSCH provides valuable national and state-level data for analyzing trends and informing healthcare research. To ensure accuracy and representativeness, the NSCH employs a rigorous quadrinomial sampling approach, incorporating stratified, multi-stage sampling and calibrated sampling weights. These weights adjust for selection probabilities, non-response, and demographic factors such as race/ethnicity and poverty level using data from the American Community Survey (ACS). Stratification enhances the reliability of survey estimates by ensuring proportional representation across different population segments, thereby reducing sampling variance. To address missing data, prevalence estimates exclude incomplete responses, while critical variables, such as poverty level, are imputed using multiple techniques to improve data completeness and reduce bias (Child and Adolescent Health Measurement Initiative [CAHMI], 2023).

The NSCH 2021 dataset was selected for this study due to its balance between brevity and validity, particularly in assessing young children’s social and emotional development (Ghandour et al., 2024). Recent efforts have sought to refine NSCH measures—such as self-regulation, emotional skills, and school readiness—through exploratory and confirmatory analyses (Ghandour et al., 2019, 2021). These ongoing pilot studies continue to enhance the survey’s reliability and impact, reinforcing its importance as a key resource for monitoring and researching children’s behavioral responses. Additionally, the dataset provides a meaningful reference for interventions designed to address the diverse needs of families (Ghandour et al., 2024; U. S. Department of Commerce and U.S. Census Bureau, 2023). As suggested by researchers (Zarei et al., 2023), the NSCH dataset includes parent-reported data on children, offering valuable insights into parenting. Leveraging contextual constructs from the dataset can deepen our understanding of the social–emotional mechanisms underlying children’s behaviors.

### 1.5 The present study

**Figure 1:**
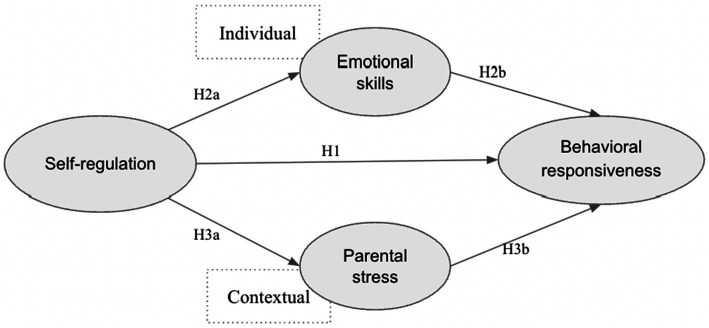
The recommended model of the study.

This study aims to investigate whether parental stress (contextual) and emotional skills (individual) mediate the relationship between self-regulation and children’s behavioral responsiveness in challenging preschool situations. The study contributes to the literature by examining contextual and individual factors simultaneously to test mediating pathways, utilizing the NSCH 2021 dataset with a nationally representative sample. Understanding the mechanisms through which self-regulation influences behavioral responsiveness in preschool children is crucial for effectively supporting individual children, preparing families and schools to provide appropriate services, and designing tiered intervention programs. These programs include tier 2 and tier 3 interventions for at-risk groups, in addition to tier 1 universal instruction for all age-appropriate children. The conceptual relationship of the proposed model is presented in Figure 1.

## 2 Method

### 2.1 Study sample

This study utilized data from the 2021 National Survey of Children’s Health (NSCH) to examine the relationship between children’s self-regulation and behavioral responsiveness among young children in the United States. The 2021 NSCH surveyed 50,892 children aged 0–17 years. This study focused specifically on children aged 3 to 5 years, analyzing data from 11,554 participants within this age group.

Among these children, 52.2% were male-identified. The sample’s demographic composition included 13.21% Hispanic children, 65.97% non-Hispanic White children, 6.17% non-Hispanic Black or African American children, and 14.66% from other racial or multi-racial backgrounds. Additionally, over 8% of children lived in households where languages other than English were spoken, 10% were identified as having a disability, and nearly 40% lived in households with incomes at or below 200% of the federal poverty line.

The NSCH is an annual, nationally representative survey designed to assess various aspects of children’s health, well-being, and development, along with their family and community environments at both national and state levels. The survey follows a two-step process: first collecting basic demographic information, then administering a questionnaire to a parent or guardian of the selected child, covering topics related to the child’s health, development, and family context.

### 2.2 Measures

Originally, the dataset used a scale from 1 (“Always”) to 5 (“Never”) for parent or guardian ratings. Before analysis, all negatively worded items were reverse-coded so that “Always” corresponded to a value of 5, ensuring higher values represented stronger levels of the construct. Additionally, any omitted or invalid responses (denoted by a dot in the original dataset) were treated as missing values. Item selection was based on theoretical alignment, results from confirmatory factor analysis (CFA), and consistency with previous NSCH pilot measure studies (e.g., Ghandour et al., 2019; Webster et al., 2025). Specifically, items were structured to align with variables from those prior studies, ensuring psychometric soundness and factor loadings that fit well within the proposed model (Figure 1). The demographic control variables included biological sex (male coded as 1), age (3–5 years), and family poverty status (coded based on whether household income was above or below 200% of the federal poverty level).

#### 2.2.1 Independent variable

Self-regulation, also referred to as self-control or self-management, involves the intentional use of one’s abilities to appropriately respond to environmental demands (Kochanska et al., 2001; Ponitz et al., 2008, 2009). This study followed previous NSCH pilot measures of self-regulation in parent reports (Ghandour et al., 2019; Webster et al., 2025), incorporating items such as: “Compared to other children their age, how often is this child able to sit still?”; “How often does this child keep working at something until they are finished?”; and “When this child is paying attention, how often can they follow instructions to complete a simple task?” After analyzing these items, three items were selected for the final measure of self-regulation (Cronbach’s alpha = 0.7), which reflects parents’ perceptions of children’s self-regulation, based on CFA results (see Table 1).

**Table 1:** Items and factor loadings in the corresponding constructs.

Latent variables	Observed items in survey	Label in NSCH	Factor loadings	Reliability	Fit indices
Self-regulation	Compared to other children their age, how often is this child able to sit still?	SITSTILL	0.61	0.7	CFI: 0.99 TLI: 0.99 SRMR:0.01
	How often does this child keep working at something until they are finished?	WORKTOFIN	0.64		
	When this child is paying attention, how often can they follow instructions to complete a simple task?	SIMPLEINST	0.61		
Parental stress	During the past month, how often have you felt that this child is much harder to care for than most children his or her age?	K8Q31	0.70	0.8	CFI: 0.99 TLI: 0.99 SRMR:0.01
	During the past month, how often have you felt that this child does things that really bother you a lot?	K8Q32	0.84		
	During the past month, how often have you felt that you feel angry with this child?	K8Q34	0.66		
Emotional skills	Is this child affectionate and tender with you?	K6Q70_R	0.59	0.7	CFI: 0.97 TLI: 0.91 SRMR:0.03
	Does this child smile and laugh?	K6Q71_R	0.60		
	Does this child bounce back quickly when things do not go their way?	K6Q72_R	0.62		
	Does this child show interest and curiosity in learning new things?	K6Q73_R	0.58		
Behavioral responsiveness	How often does this child become angry or anxious when transitioning between activities?	NEWACTIVITY	0.64	0.7	CFI: 0.99 TLI: 0.99 SRMR:0.01
	How often does this child lose control of their temper when things do not go their way?	TEMPER	0.63		
	How often is this child easily distracted?	DISTRACTED	0.62		

#### 2.2.2 Mediator variable (context)

In the NSCH dataset, parental stress was initially labeled as Parental Aggravation (ParAggrav_21) in the measurement instrument codebooks (Child and Adolescent Health Measurement Initiative [CAHMI], 2023). However, due to more common usage of the term parental stress in prior research (e.g., Campisi et al., 2024; Crouch et al., 2024; Streisand et al., 2010; Tan et al., 2017), this terminology was adopted for consistency. The items measuring parental stress included: “During the past month, how often have you felt that this child is much harder to care for than most children his or her age?”; “This child does things that really bother you a lot?”; and “You feel angry with this child?” The internal consistency for parental stress was 0.8, indicating good reliability and suggesting the items measure the same construct.

#### 2.2.3 Mediator variable (individual)

Following a similar selection process as parental stress, four items were used to assess children’s emotional skills, aligned with CFA and previous NSCH pilot studies (Ghandour et al., 2019; Webster et al., 2025): “Is this child affectionate and tender with you?”; “Does this child smile and laugh?”; “Does this child bounce back quickly when things do not go their way?”; and “Does this child show interest and curiosity in learning new things?” The reliability for emotional skills was 0.7, suggesting acceptable internal consistency.

#### 2.2.4 Dependent variable

The dependent variable in this study, behavioral responsiveness, measured preschoolers’ reactions to challenging, frustrating, and distracting situations, which are critical for children’s behavioral development. Three items were constructed based on CFA, including: “How often does this child become angry or anxious when transitioning between activities?”; “How often does this child lose control of their temper when things do not go their way?”; and “How often is this child easily distracted?” The reliability for behavioral responsiveness was 0.7, indicating acceptable internal consistency. Although similar constructs are sometimes labeled as problem behaviors (e.g., Anderson et al., 2024; Lent et al., 2024), we define these behaviors as children’s behavioral responses to unpleasant situations. Given the developmental stage of the sample (aged 3–5 years), labeling them as problem behaviors would be premature, as this stage is crucial for their behavioral growth and learning.

### 2.3 Analysis

**Figure 2:**
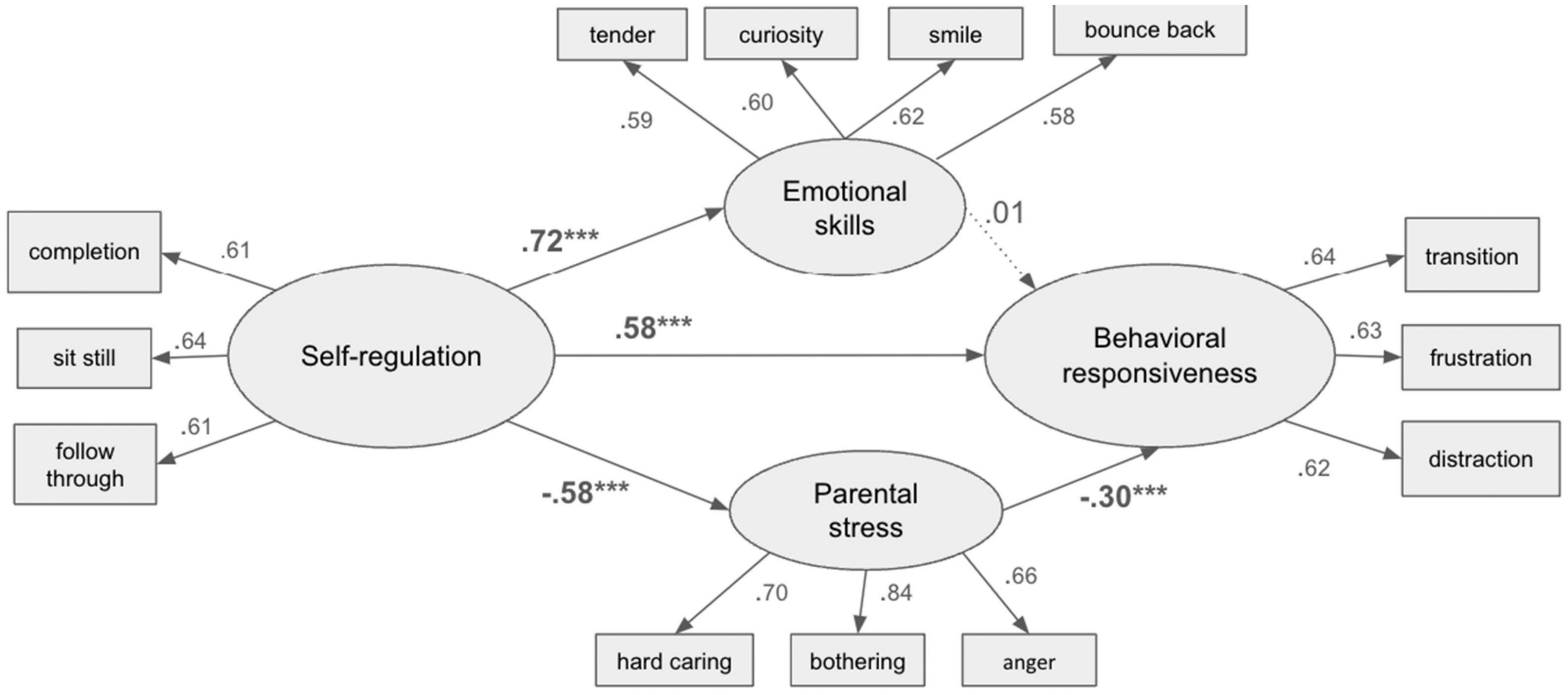
Full structural equation model. *p < 0.05, **p < 0.01, ***p < 0.001.

The analyses were conducted using *Stata 17.0*, which employs maximum likelihood to estimate the structural equation modeling (SEM). Preliminary descriptive statistics and correlation assessments were tested (see Table 2). The initial step involved conducting CFAs to determine whether self-regulation, parental stress, emotional skills, and behavioral responsiveness could be distinct factors, with low factor loading items being excluded from the analysis. The structural equation model was then conducted to examine the mediating effect of contextual parental stress and individual emotional skills on the relationship between children’s self-regulation and behavioral responsiveness (see Figure 2), which elucidate the mechanism between exogenous and endogenous variables through the inclusion of mediators. Children’s behavioral responsiveness factor was regressed on self-regulation, parental stress and children’s emotional skills. Socioeconomic status (SES), gender, and age were statistically controlled to reduce the potential influence of background contextual factors on the primary associations of interest, allowing for a more precise estimation of the indirect effects through the proposed mediators. According to guidelines, we used the following indices for model fit: comparative fit index (CFI) > 0.90, root mean-square-error of approximation (RMSEA) < 0.08, and standardized root mean-square-residual (SRMR) < 0.06 (Hair et al., 2021). We used the following syntax to account for design effects and sampling weights issues, “svyset hhid [pw = fwc], strata(stratacr),” “svy, subpop(select).” Missing data were handled by maximum likelihood estimation with robust standard errors and chi-square calculation in the presence of missing values as there was only a small fragment of missing data. The following guidelines were used to interpret the magnitude and relevance of the standardized beta coefficients: less than 0.05 is small, 0.05 to less than 0.20 is medium, and 0.20 or greater is large (Kraft, 2020).

**Table 2:** Descriptive statistics and correlations for all item-level measures.

Measures	1	2	3	4	5	6	7	8	9	10	11	12	13
1. SR – completion	–												
2. SR – sitstill	0.38	–											
3. SR – followthrough	0.39	0.36	–										
4. ES – tender	0.20	0.22	0.20	–									
5. ES – curiosity	0.26	0.23	0.27	0.31	–								
6. ES – bounceback	0.15	0.15	0.16	0.39	0.34	–							
7. ES – smile	0.23	0.29	0.25	0.34	0.32	0.27	–						
8. PS – hard caring	−0.27	−0.35	−0.28	−0.27	−0.23	−0.21	−0.32	–					
9. PS – bothering	−0.23	−0.28	−0.22	−0.29	−0.21	−0.21	−0.30	0.57	–				
10. PS – anger	−0.18	−0.21	−0.14	−0.24	−0.15	−0.17	−0.23	0.42	0.61	–			
11. BR – transition	−0.23	−0.28	−0.27	−0.22	−0.21	−0.18	−0.28	0.34	0.31	0.24	–		
12. BR – frustration	−0.23	−0.29	−0.21	−0.20	−0.17	−0.14	−0.30	0.36	0.35	0.30	0.41	–	
13. BR – distraction	−0.33	−0.40	−0.28	−0.15	−0.18	−0.09	−0.20	0.31	0.27	0.20	0.34	0.36	–
*M*	3.55	3.69	4.23	3.64	3.46	3.21	3.80	1.61	1.82	1.84	1.87	2.40	2.51
*SD*	0.86	0.88	0.78	0.56	0.71	0.69	0.43	0.90	0.83	0.73	0.77	0.88	0.89
Skewness	−0.64	−0.72	−1.17	−1.38	−1.07	−0.53	−2.02	1.518	0.750	0.435	1.279	0.948	1.062
Kurtosis	0.10	0.27	1.88	1.64	0.30	0.02	3.96	1.86	0.08	−0.42	2.91	0.65	0.72

SR, self-regulation; ES, emotional skills; PS, parental stress; BR, behavioral responsiveness; M, mean; SD, standard deviation.

## 3 Results

### 3.1 Measurement models

Descriptive statistics including mean scores, standard deviations, and correlations for each measure are presented in Table 2. The measurement models with CFA demonstrated acceptable model fits, with all CFI and TLI values above 0.90 and SRMR values below 0.04. Table 1 presents the factor loadings for each item and the reliability for the variables. Both the fit indices and the strong internal consistency suggested that the latent constructs were distinct factors.

### 3.2 SEM results

We estimated the Structural Equation Model (SEM) shown in Figure 2, which operationalized the model depicted in Figure 1. The fit statistics for the SEM were as follows: RMSEA = 0.07, CFI = 0.91, and SRMR = 0.05, indicating an acceptable fit to the data. The standardized path coefficients for the SEM are presented in Table 3.

**Table 3:** Path coefficients and *z*-scores for the SEM.

Factors	Path coefficient	*z*-score
Self-regulation → Behavioral responsiveness	0.58***	0.12
Parental stress → Behavioral responsiveness	−0.30***	0.05
Emotional skills → Behavioral responsiveness	0.01	0.02
Self-regulation → Parental stress	−0.58***	0.03
Self-regulation → Emotional skills	0.72***	0.03

***p < 0.001.

The findings revealed that children’s self-regulation was the strongest predictor of their behavioral responsiveness (*ꞵ* = −0.58, *p* < 0.001), after controlling for demographic variables. Specifically, a one standard deviation (SD) increase in children’s self-regulation was associated with a 0.58 SD increase in behavioral responsiveness, meaning children exhibited better responses to transitions, frustrations, and distractions. Conversely, parental stress was a significant and positive predictor of behavioral responsiveness (*ꞵ* = 0.30, *p* < 0.001). A one SD increase in parental stress was associated with a 0.30 SD decrease in children’s behavioral responsiveness, suggesting that higher parental stress was linked to poorer behavioral responses in children. Notably, emotional skills did not show a significant effect on children’s behavioral responsiveness within the SEM.

Additionally, the analysis revealed strong and significant relationships between the mediators and self-regulation. Specifically, a one SD increase in children’s self-regulation was associated with a 0.72 SD increase in emotional skills (*p* < 0.001) and a 0.58 SD decrease in parental stress (*p* < 0.001). These results indicated a mediating effect of contextual parental stress in the relationship between self-regulation and children’s behavioral responsiveness. For a full summary of the SEM path coefficients, refer to Table 3.

## 4 Discussion

This study investigated how both individual and contextual factors mediate the relationship between children’s self-regulation and their behavioral responsiveness to challenging, frustrating, and distracting situations, with a particular focus on the affective mechanisms involving parental stress and children’s emotional skills. This investigation was particularly pertinent given the increasing recognition of the ecological factors affecting child development.

Initially, we found that children’s self-regulation significantly and positively influenced their appropriate behaviors and reactions in the face of transition, frustration, and distraction, which supports hypothesis H1 and is consistent with existing literature (Eisenberg et al., 2017; Sulik et al., 2015). It indicates that higher levels of self-regulation are associated with better behavioral responsiveness, with a substantial effect size (over 0.5). Children demonstrating lower levels of self-regulation were more prone to engaging in off-task behaviors during instructional activities and learning, particularly when confronted with challenging tasks (Rimm-Kaufman et al., 2005). This highlights the importance of children’s capacity to regulate thoughts and attention, as it aids in a child’s ability to regulate their behavioral responses to contextual stimuli (Blair and Raver, 2015). This finding was especially encouraging, as the skills children acquired in preschool, particularly self-regulation, are crucial for their learning and behavior required for formal schooling when they enter kindergarten. Therefore, understanding how early self-regulation is linked to children’s behavioral responsiveness is essential for fostering the positive development of these skills in the classroom.

Additionally, the effect of children’s self-regulation on their behavioral responsiveness was significantly mediated by parental stress. The study demonstrated robust connections among children’s self-regulation, parental stress, and behavioral responsiveness. Specifically, there was an inverse correlation between self-regulation and parental stress, and an inverse correlation between parental stress and behavioral responsiveness in transition, frustration, and distraction, indicating children with a higher level of self-regulation tended to lessen their parents’ stress in caregiving, which in turn facilitated children’s behavioral responsiveness under unpleasant contexts. This outcome, which supports hypothesis H3, aligned with the findings of Chiang and Bai (2024) and emphasized the mediating role of a lower level of parental stress in the relationship between self-regulation and appropriate behaviors. Anxious parents often impose strict control over their children’s learning and daily activities, which can hinder self-development, exacerbate negative behaviors, and lead to an increase in inappropriate responses in transition. Extensive research supports this view, showing a negative correlation between children’s behavioral responsiveness and parental stress, such as caregiver anxiety (Clauser et al., 2021), and a negative correlation with self-regulation, such as perseverance in learning. This suggests that children’s behaviors are influenced not only directly by their self-regulation abilities but also indirectly through the impact of parental stress on parenting practices. Researchers have demonstrated that a lack of behavioral responsiveness is associated with parenting conflicts and strained parent–child relationships (Chiang and Bai, 2024; Crum and Moreland, 2017). Children who showed a lack of behavioral responsiveness during transitions, frustrations, and distractions were more prone to facing rejection and disciplinary actions from their parents, which reduced their chances of engaging in positive behaviors. According to Montroy et al. (2014), children exhibiting lower levels of self-regulation were observed to display increased impulsivity and were perceived to engage in bullying behavior more frequently compared to children with higher self-regulation levels. Likewise, our structural equation modeling findings suggested that a pathway through which self-regulation related to children’s behavioral responsiveness was by contextually alleviating stress in parenting, leading to the promotion of appropriate behaviors (e.g., control of their temper when things do not go their way).

Lastly and notably, the study examined the concurrent mediating role of individual emotional skills in the connection between self-regulation and behavioral responsiveness, in order to test hypothesis H2. Contrary to our expectations, emotional skills did not significantly mediate the relationship between self-regulation and behavioral responsiveness, while the connection between children’s self-regulation abilities and emotional skills was positive and significant with a large effect size. This indicated that when included together in a multiple regression analysis, children’s self-regulation abilities and emotional skills showed the strongest relationship, whereas emotional skills did not act as a significant mediator. This further suggested that when both factors were considered together in the mediation regression, the mediating effects worked through contextual factors (i.e., parental stress) rather than individual factors (i.e., emotional skills). One possible explanation is that emotional skills, while related to self-regulatory capacity, may exert a more distal or context-dependent influence on children’s behavioral outcomes compared to more proximal environmental factors such as parenting stress. Prior research has shown that emotional understanding and empathy may be more strongly linked to social competence or prosocial behavior (e.g., Bierman et al., 2008; Denham et al., 2003) than to the specific behavioral responsiveness outcomes assessed in this study. Additionally, the measurement of emotional skills in this dataset may have captured trait-level dispositions rather than situational processing or application, which could limit its role as a dynamic mediator in the model.

Collectively, our findings provided further support for the underlying pathways to children’s behavioral responsiveness to transitions, frustrations, and distractions, and could guide the development of prevention and early intervention programs that focus on contextual factors, such as parenting, rather than merely individual factors. Strategies of positive parenting alleviate stress in parent–child interactions, through which altering children’s responses to situations through behaviors and thoughts. Notably, our focus was on the parenting journey, particularly the impact of parental stress, rather than on controlling every behavior of the child. We recognized that preschoolers are still developing self-regulation and may occasionally struggle with emotional control. However, our findings underscore the comparatively stronger influence of contextual factors, particularly parental stress, over individual-level traits like emotional skills in shaping behavioral responsiveness during early childhood. This highlights the foundational role of the caregiving environment in supporting children’s development and future outcomes.

## 5 Implications and limitations

This research reveals the underlying mechanisms affecting children’s behavioral responsiveness, providing significant theoretical and practical insights. Theoretically, it explored the roles of individual versus contextual mediators by comparing the impacts of both children’s and parents’ emotional factors. This analysis differed from other studies that concentrated exclusively on various mediators at the same level. Despite growing recognition of the interaction with family dynamics, the complex pathways linking children’s self-regulation to their behavioral responses remained less clear. By exploring both individual and contextual mediators, a more comprehensive perspective emerged, highlighting the distinct roles of children’s emotional skills and parental stress. The study clarified the comparative impact of contextual factors on children’s development in relation to individual skills.

Practically, understanding how contextual parental stress mediates children’s self-regulation and behavioral responsiveness offered crucial insights for designing intervention programs that help parents reduce their children’s inappropriate behaviors. The stress experienced by parents during caregiving and the parenting process should not be overlooked. Recent data showed that over 30% of parents indicated experiencing significant stress in the past month due to childcare issues (Lebrun-Harris et al., 2022). Understanding the strong role of parental stress as a mediator was vital for improving caregiving methods, creating supportive family environments, and advancing evidence-based parenting practices, all of which contribute to positive child behaviors. Elevated levels of children’s self-regulation enabled children to effectively and comfortably interact with their parents, which in turn fostered their appropriate behavioral responsiveness to transitional, frustrating, and distracting situations. This study proposed strategies to reduce negative behaviors in preschoolers by focusing on decreasing parental stress in parenting approaches.

Additionally, the findings of the current study suggested the importance of promoting children’s self-regulation from an early age. Self-regulation allows young children to manage or inhibit automatic or ingrained responses, typically to pursue goal-directed actions (Lonigan et al., 2017). Research has consistently demonstrated that children exhibit improved academic performance, positive school attitudes, higher aspirations, and other favorable behaviors when they possess robust self-regulation strategies. Unfortunately, it has been observed that a significant number of preschoolers lack the necessary self-regulatory skills required for a smooth transition to kindergarten. Considering that self-regulation plays a key role in various aspects of preschool learning, it is important to develop strategies for improving early self-regulation skills within preschool classrooms for school readiness. Several curricula claim to enhance early self-regulation skills (e.g., Second Step Early Learning, CASEL), and research indicates that a curriculum including activities specifically designed to target self-regulation is linked to notably improved performance on self-regulatory tasks in preschool (Ross and Tolan, 2018). Future research on the effectiveness of self-regulation training is necessary to better understand how to support young children’s preschool learning and encourage appropriate behaviors through improved self-regulation.

Several limitations of the current study should be taken into account. The current study’s measures were based on parent reports, consistent with methodologies used in previous research in this field (Bethell et al., 2017). Parent reports can reflect both the child’s situational patterns and the parent’s perceptions of the child, which may be subject to bias. The inclination to view successful parenting as socially desirable can influence parents’ responses, potentially causing discrepancies between their assessments and their children’s actual experiences, thereby introducing statistical noise into the data. Additionally, the measurement, consistent with prior research (Ghandour et al., 2019; Webster et al., 2025), included a limited number of items because of the national secondary dataset, which might limit the validity of the construct. For instance, although parental stress was assessed through caregivers’ self-reported attitudes toward the challenges of parenting, this measure captures only one dimension of broader parenting attitudes. The absence of a more comprehensive assessment of parenting beliefs and values limits our ability to examine how different facets of parenting attitudes may interact with stress and influence child outcomes. An important direction for future research would be to examine this issue using observational measures, taking into account multidimensional measures to more fully capture the roles in parent–child dynamics. Next, the study relied on cross-sectional data and did not track children through their transition developmentally, limiting our ability to assess whether these relationships evolved or persisted over time. Future research should employ longitudinal data to explore these processes over time or to test cross-lagged reciprocal relationships among the factors.

## 6 Conclusion

The present study adds to the literature by examining the relationship between children’s self-regulation, emotional skills, parental stress, and behavioral responsiveness in preschool children. A high level of self-regulation was closely associated with improved emotional skills in children, reduced parental stress, and better behavioral responsiveness to transitions, frustrations, and distractions. Our findings, based on the NSCH 2021 dataset featuring a nationally representative sample, corroborated existing literature indicating that when regressed together, contextual parental stress mediated the relationship between self-regulation and behavioral responsiveness, whereas children’s emotional skills did not. This finding urged policymakers to include parent coping strategies in the program design and implementation. Researchers and educators need to thoughtfully explore the best strategies for supporting self-regulatory skills in the classroom and providing intervention programs for positive parenting to lessen parental stress and ultimately assist children in cultivating appropriate behaviors when they encounter transitions, frustrations, and distractions.

## Data Availability

The data used in this study are publicly available from the 2021 National Survey of Children’s Health (NSCH), which is administered by the U.S. Census Bureau and sponsored by the Health Resources and Services Administration (HRSA). The dataset can be accessed through the NSCH Data Resource Center upon registration: https://www.childhealthdata.org/. No personally identifiable information is included in the public-use dataset. Further inquiries can be directed to the corresponding author.
